# Paediatric palliative home care by general paediatricians: a multimethod study on perceived barriers and incentives

**DOI:** 10.1186/1472-684X-9-11

**Published:** 2010-06-04

**Authors:** Saskia Jünger, Andrea E Vedder, Sigurd Milde, Thomas Fischbach, Boris Zernikow, Lukas Radbruch

**Affiliations:** 1 Department of Palliative Medicine, RWTH Aachen University Hospital, Pauwelsstraße 30, 52057 Aachen, Germany; 2 Berufsverband der Kinder- und Jugendärzte, Landesverband Westfalen-Lippe, Germany; 3 Berufsverband der Kinder- und Jugendärzte, Landesverband Nordrhein, Germany; 4 Vodafone Foundation Institute and Chair of Children's Pain Therapy and Paediatric Palliative Care, Clinic for Children and Adolescents Datteln, Dr.-Friedrich-Steiner-Straße 5, 45711 Datteln, Germany

## Abstract

**Background:**

Non-specialist palliative care, as it is delivered by general practitioners, is a basic component of a comprehensive palliative care infrastructure for adult patients with progressive and far advanced disease. Currently palliative care for children and adolescents is recognized as a distinct entity of care, requiring networks of service providers across different settings, including paediatricians working in general practice. In Germany, the medical home care for children and adolescents is to a large extent delivered by general paediatricians working in their own practice. However, these are rarely confronted with children suffering from life-limiting diseases. The aim of this study was therefore to examine potential barriers, incentives, and the professional self-image of general paediatricians with regard to paediatric palliative care.

**Methods:**

Based on qualitative expert interviews, a questionnaire was designed and a survey among general paediatricians in their own practice (n = 293) was undertaken. The survey has been developed and performed in close cooperation with the regional professional association of paediatricians.

**Results:**

The results showed a high disposition on part of the paediatricians to engage in palliative care, and the majority of respondents regarded palliative care as part of their profile. Main barriers for the implementation were time restrictions (40.7%) and financial burden (31.6%), sole responsibility without team support (31.1%), as well as formal requirements such as forms and prescriptions (26.6%). Major facilitations were support by local specialist services such as home care nursing service (83.0%), access to a specialist paediatric palliative care consultation team (82.4%), as well as an option of exchange with colleagues (60.1%).

**Conclusions:**

Altogether, the high commitment to this survey reflects the relevance of the issue for paediatricians working in general practice. Education in basic palliative care competence and communication skills was seen as an important prerequisite for the engagement in paediatric palliative home care. A local network of specialist support on site and a 24/7 on-call service are necessary in order to facilitate the implementation of basic palliative care by paediatricians in their own practice.

## Background

In the German health care system primary care for children, especially for babies and infants, is usually provided by paediatric specialists working in general practice, even if it may also be provided by a general practitioner. Correspondingly, there is a high density of paediatricians working in general practice in Germany. In North Rhine-Westphalia there are approximately 1.300 paediatricians working in general practice for a population of 3.6 million children and adolescents. Consequently, setting up networks for palliative care for children and adolescents, providing continuous care throughout a wide range of settings, the paediatrician in general practice comes into the focus as one of the main stakeholders in paediatric home care. However, to this date the provision of palliative home care by general paediatricians is not self-evident. In the course of their professional activity general paediatricians are only rarely confronted with the need of palliative care for one of their patients. In consequence, they do not have experience in dealing with this issue [1,2]. Moreover, palliative care needs in children are heterogeneous; and according to the respective diagnosis and illness trajectory different approaches and measures are indicated [3-6]. It is not feasible for a generalist to be familiar with all these particular care demands. Uncertainties among general paediatricians with regard to communication with patient and family, do not resuscitate status, pain management and the transition from curative to palliative care have been described in recent studies [1,2,7]. In addition, structural barriers within the health care system can play a role. For example, there is a lack of social counselling in the home care setting, and general paediatricians often lack knowledge about which medical supplies are needed and how they can be applied for. Special prescription forms for opioids have to be ordered by the individual physician and are not always on hand. Remuneration issues, organisational barriers like huge distances in rural areas, as well as questions of responsibilities and referral at the interface between inpatient and outpatient sector can complicate the involvement of generalists in paediatric palliative home care [8,9].

The lack of experience and practical obstacles can be reinforced by emotional barriers [1,7,10]. Many paediatricians do not feel comfortable with the care for dying children.

Despite these difficulties, the federal association of paediatricians in North Rhine-Westphalia regards the care for severely ill and dying children as an original task of paediatricians in general practice. Also international paediatric and palliative care professional associations recommend that all paediatricians should be familiar and comfortable with the basic principles of palliative care for children and adolescents [11,12].

In North Rhine-Westphalia, a federal state in the North-West of Germany, a pilot project was implemented in 2007 for the development and implementation of paediatric palliative home care services [13]. North Rhine-Westphalia has an area of 34.088,31 km² with 17.996.621 inhabitants (population density: 528 inhabitants/km²), among which 3.662.949 children. For the purpose of the pilot project, two specialist centres for paediatric palliative care have been set up in the two regions North Rhine and Westphalia-Lippe, each with a multiprofessional team of nurses, physicians, and psychosocial staff. Their task is the coordination of existing services and the establishment of regional care networks, as well as consultation and provision of specialist care for children and adolescents with a life-limiting condition. Evaluation of the project included the description of the status quo of service delivery at the outset of the pilot project, as well as the evaluation of the activities of the specialist centres throughout the project period. Within the framework of the development of a network for integrative palliative home care for children and adolescents, it is therefore pivotal to explore the barriers and incentives related to the involvement of generalist paediatricians in palliative care.

## Methods

A two-step approach was chosen to investigate this issue. First, a qualitative explorative part was used to identify topics and generate research questions. Secondly, a questionnaire survey was undertaken to study the core issues emerging from part I in a broader sample.

### Step I: Qualitative exploration

For the first exploration, semi-structured interviews with paediatricians in general practice (n = 5) were undertaken.

### Procedure

These paediatricians have been approached as part of a larger interview survey among experts from the fields of paediatrics, palliative care and hospice care to get access to specialist knowledge and experience with respect to the subject in question [14]. In accordance with the explorative nature of this study, the interview partners were selected by means of convenience sampling; they have been recommended by paediatric palliative care specialists who knew them from previous collaboration. The interviews (n = 5) were conducted using a semi-structured interview guide and led in an open, explorative way that allowed for themes to emerge based on the individual experiences of the interview partner. The paediatricians were invited to talk about their experiences with paediatric palliative care. They were asked for specific care situations which were an example of well-organised care delivery, as well as for examples which in contrast illustrated shortcomings and gaps within the existing care delivery. In addition, issues that had emerged from previous literature search were actively enquired such as their attitude towards paediatric palliative care, how sure they felt about prognosis in children with life-limiting diseases, or the compatibility of the care for a dying child with the patient load in their general practice. The interviews were accomplished in the experts' work environment. At the outset of each interview, informed consent was obtained. The interviews were conducted by a trained interviewer (SJ) and lasted between 30 and 120 minutes with an average length of 90 minutes. All interviews were digitally recorded and transcribed verbatim.

### Data analysis

Qualitative content analysis was chosen as the most suitable method for a data analysis as an inductive approach that at the same time follows theoretical guidelines [14,15]. The analysis was supported by a software programme for text analysis (MAXQDA^®^). Transcripts were initially free-coded by the interviewer (SJ) according to content, and then organised into thematic units that were continually re-visited and revised. A code system with key categories was developed by the interviewer (SJ) and revised by two independent coders experienced in qualitative text analysis (MP, TP). In addition to the findings from the previous literature search, the categories that emerged from the expert interviews allowed for refining the following research questions (for details see results of the qualitative exploration):

1. How often are paediatricians in their own practice confronted with the demand for palliative care for their patients?

2. What are incentives and facilitations for the implementation of paediatric palliative care in the paediatricians' view?

3. Which are the main barriers for the implementation of palliative home care for children and adolescents?

4. Which kind of support do paediatricians in general practice need in order to participate in the provision of palliative care in terms of

▪ skills and knowledge

▪ practical/organisational/financial facilitations

▪ emotional security.

### Step II: questionnaire survey

A standardised questionnaire has been developed on the basis of the statements in the expert interviews, as well as additional insight in the recent literature [7-9]. For an unambiguous understanding, the WHO-definition of paediatric palliative care [16] was provided at the beginning of the questionnaire (Additional file 1). Moreover, participants were informed about the anonymous processing of their responses. Ethical approval for the study was given by the Ethics Committee of the RWTH Aachen University Hospital.

The questionnaire comprises six thematic areas:

▪ previous experience with paediatric palliative care

▪ disposition to provide palliative care for children and adolescents

▪ perceived barriers in the implementation

▪ facilitations and incentives for the implementation

▪ professional role of the general paediatrician within the paediatric palliative care network

▪ demographical data.

**Figure 1:**
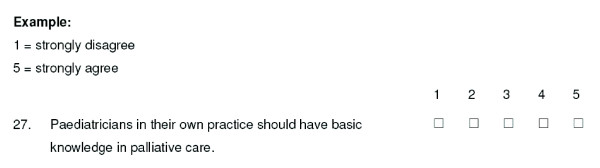
Example questionnaire item

The questions on barriers and facilitations, as well as on the professional role, consist of statements with a homogeneous item- and response format (five-point or six-point Likert scales; Figure 1).

An opportunity for additional free text responses was provided. The questionnaire was pilot-tested during a quality circle for paediatricians with n = 14 participants by means of think-aloud-testing [17,18]. The questions were answered per thematic area; incomprehensibilities were immediately clarified. Despite this extended procedure the completion of the questionnaire took less than 15 minutes. The pilot test revealed that the instructions for completion were clear, and that the response options in closed questions sufficiently covered a relevant range of alternatives. The length and the duration were perceived as reasonable, and the wording of the questionnaire adequately corresponded with the sensible subject. A differentiated spectrum of answers could be denoted. Based on the results of the pilot-test, technical errors in the lay-out were solved and mistakable wordings were adapted. Two questions were added (need for further education and 24/7 on-call service).

### Procedure

The survey was undertaken between April 2008 and October 2008; it was conducted in close cooperation with the federal association of paediatricians. Since every paediatrician is supposed to participate in a regional quality circle in order to gather the required credit points for continuing education - which are a prerequisite to maintain their accreditation - this method ensures a representative selection of respondents for the present survey. The chairmen of the associations in North Rhine and in Westphalia-Lippe were involved in the development of the questionnaire and the procedure itself in order to warrant a high practical relevance of the survey. The survey was performed during the regional quality circles to reach a response as high as possible with minimum effort for the participants. The associations' chairmen informed the regional speakers of the paediatric quality circles about the study by providing explicit approval for participation in the survey on part of the federal association.

Based on a total number of approximately 1.300 paediatricians working in their own practice in North Rhine-Westphalia, the aim was to reach a representative sample of n = 300. The quality circles were approached by means of judgement sampling. This is a non-probability sampling method where the researcher decides which population members to include in the sample based on his judgement providing justification for the representativeness of the sample. In the present study, the quality circles have been selected taking account of a representative distribution of urban and rural areas, as well as from the two regions within North Rhine-Westphalia, that is North Rhine and Westphalia-Lippe. In a first step, 16 regional districts in rural and urban areas were selected and the respective speakers were contacted by the principal investigator (SJ) by phone. In a second step, further districts were selected until the aspired sample size was reached.

If possible, either the principal investigator (SJ) or the research assistant (AV) personally attended the quality circles in order to point out the aims of the research, answer questions, and collect the questionnaires afterwards. In some cases the questionnaires were sent to the regional speaker with a self-addressed stamped envelope and an information letter.

### Data analysis

Descriptive analyses were performed with the statistical package for social sciences SPSS 16.0. For the calculation of percentages regarding the perceived barriers and incentives, the 5-point Likert scales were recoded into dichotomous variables (values 1 - 3 were combined to "is not perceived as a relevant barrier/incentive"; values 4 and 5 were summarized as "is perceived as a relevant barrier/incentive"). For comparisons between groups, X^2^-tests were used.

## Results

### Qualitative exploration

Six main categories emerged from the data (Table 1): prior experiences with palliative care, emotional attitude towards palliative care, barriers (emotional, professional, structural), facilitations, and the paediatricians' role and professional self-image.

**Table 1:** Palliative home care by general paediatricians - issues emerged from the expert interviews

Prior experiences with palliative care	Emotional attitude towards palliative care	Barriers	Incentives/facilitations	Disposition for palliative care	Role of the general paediatrician in palliative care
First contact with palliative care		Emotional barriers	Network/collaboration		Character and duration of the care
Frequency in daily practice	No subcategories	Structural barriers	Emotional facilitations	No subcategories	Key contact person
Circumstances		Professional barriers	Structural facilitations		Transition from curative to palliative
		Reluctance on part of the parents	Professional facilitations		Palliative care is part of medical practice

#### Prior experiences with palliative care

It became clear from the interviews that the need for palliative care in the paediatricians' daily practice occurs only rarely. The interview partners on average reported 1 case per year or less.

#### Emotional attitude towards palliative care

During the interviews, the paediatricians reflected their emotional attitude towards the care for a dying child, as the following quote illustrates.

"And than, at a certain point, I have to take the decision: what can I do as a physician for this person, instead of talking about dosage, the drip, or whatever, these whole medical bells and whistles. At a point when death is approaching, all of this is pretty relative. And either I decide to remain reachable for this patient - also in a metaphoric sense - or internally I have already logged out."

#### Barriers

The lack of experience and routine was described as the major reason for professional barriers. Structural barriers mainly originated from the general conditions of the health care system, for example inadequate remuneration, organisational obstacles such as long distances, as well as questions of responsibility, referral, and the interface between inpatient and outpatient care.

#### Facilitations

Confidence on part of the patient and the family was seen as an important facilitation for the paediatrician's work. The paediatricians mentioned the burden of the confrontation with a dying child but at the same time were confident that they will be able to care for a child with a life-limiting disease in an intuitive and natural way. The most important facilitations were seen in an adequate remuneration, as well as in a simplification of organisational procedures. The prominent incentive was the possibility to collaborate with a specialist centre and to consult a palliative care specialist.

"In this situation, when you are outside there caring for this child as the general paediatrician, you need to be able to fall back on any structure of people who are even engaged and care for this child just like yourself. Otherwise you will not be able to do this; you will really keep staying at home in such a time. Because than you will not find the courage or the freedom for yourself to say: okay, actually now I am on holiday, I ought to go on holiday. But this is not possible, because there is this child. And you just cannot do that. You cannot do that to your family. Therefore it is important that you can fall back on someone. It does not need to be every weekend, certainly, but in certain circumstances it must be possible."

"Because I will need the support nearby. And not only mere theoretical support that you can get via a hotline. For example if I, concretely speaking, will have a problem with ventilation, with anything, I will not be able to clarify this on the phone. And I think that many colleagues are simply afraid of these situations, that they say they do not feel well versed with that and than it won't work, and they will simply need someone who will help them. And if you will not be able to reach someone, because he is not available, than I think this will be rather difficult."

#### The paediatricians' role and professional self-image

The interview partners describe their professional role as a trusted key person who accompanies the families over many years. There was agreement among the interview partners that palliative care is an essential part of medical practice, and therefore they felt committed to provide this care for those children and adolescents in need of it.

"I think that as the general paediatrician I had my own part. For example I was the one who has written the death certificate, you know? [...] And I was the one who was called at night and we have washed the child together and prepared everything. So this was my emotional part as the paediatrician."

### Questionnaire survey

#### Sample

In total, n = 296 paediatricians answered the questionnaire. N = 293 questionnaires were considered for data analysis (Table 2); three were excluded because of too many missing data. Paediatricians came from 20 different districts in North Rhine-Westphalia. The majority of questionnaires (n = 274) were completed during the regional quality circles. A small number of questionnaires (n = 19) were completed individually and sent to the authors by mail. In accordance with the urban structure of North Rhine-Westphalia, the proportion of respondents from urban areas amounts to two third of the sample (67.7%). The mean age was 50 years (range 32 - 70 years) and the majority of the respondents were men (61.0%). The median duration of clinical practice was 13 years and the mean number of patients per quarter was 1697. The demographic and clinical characteristics show a high concordance with the total population of paediatricians working in general practice in North Rhine-Westphalia, as confirmed by the chairs of the two regional associations and the statistical figures on all registered paediatricians working in general practice as provided by the respective *Kassenärztliche Vereinigung*. Therefore, the sample can be regarded as highly representative for the population of paediatricians working in general practice in North Rhine-Westphalia in terms of age, gender distribution, clinical experience, and patient load.

**Table 2:** Sample characteristics

Paediatricians in general practice in North Rhine-Westphalia (n = 293)		
**Origin of questionnaires**	Quality circle Other	274 19

**Region** (n = 223)	(rather) rural (rather) urban	72 (32.3%) 151 (67.7%)

**Practice** (n = 232)	Own practice Joint practice	139 (59.9%) 93 (40.1%)

**Number of patients/quarter**	MN (SD) Range	1697 (760) 200 - 5000

**Duration of settlement (years)**	MN (SD) Median (Range)	12.8 (7.8) 13 (0 - 35)

**Sex** (n = 287)	Men Women	175 (61.0%) 112 (39.0%)

**Age**	MN (SD)	49.7 (7.4)

### Experience with paediatric palliative care

More than half of the respondents (55.2%) had no experience with palliative care in their professional practice. The majority of those who did have experience (n = 130, 44.8%), reported that this had rarely occurred: in 47.3% only one single time, in 44.2% up to five times, and only in individual cases (n = 11; 8.5%) more than five times. More than 70% of the paediatricians with experience in palliative care provided free-text information on the children they had cared for. In total n = 174 cases were described with partly incomplete information. In n = 162 cases a primary diagnosis could be identified (Table 3). The conditions were classified according to the 4 disease groups first described by the Association for Children with Life Threatening or Terminal Conditions and their Families [4,19]:

**Table 3:** Frequency of different life-limiting conditions reported by paediatricians in general practice

GROUP	DIAGNOSES AND CONDITIONS REPORTED IN FREE-TEXT	N	%
**1**	Malignant diseases: leukaemia, medulloblastoma; brain tumours (pinealoblastoma, astrocytoma, glioblastoma); osteosarcoma; liver carcinoma; rhabdomyosarcoma; bronchopulmonary dysplasia; congenital heart defect	71	43.8

**2**	Cystic fibrosis; muscular dystrophy; severe immunodeficiency; short bowel syndrome	16	9.9

**3**	(Congenital) metabolic disorders: mucopolysaccharidosis; ceroidlipofuscinosis; adrenoleukodystrophy; mitochondropathy; neurodegenerative disease; spinal muscular atrophy; Edwards Syndrome; Morbus Alexander; metachromatic leukodystrophy; Tay-Sachs-Disease; Smith-Lemli-Opitz Syndrome; Zellweger Syndrome; SSPE	38	23.5

**4**	Cerebral palsy; psychomotoric retardation; myelomeningocele; epilepsy; holoprosencephaly; apallic syndrome; de Grouchy Syndrome; condition after brain haemorrhage; encephalopathy	37	22.8

**TOTAL**		**162**	**100**

▪ Group 1: Conditions for which potentially curative treatment fails

▪ Group 2: Conditions in which intensive treatment can be expected to prolong life but premature death is likely

▪ Group 3: Progressive conditions where treatment is exclusively palliative from the time of diagnosis

▪ Group 4:Non-progressive conditions which result in an increased susceptibility to complications and premature death.

The majority of children reported by the paediatricians suffered from a condition of group 1 (43.8%), nearly all of them related to malignant diseases, mainly brain tumours. Conditions of group 3 and 4 were reported in 20% of the cases with a broad range of different diagnoses in group 3. Half of the respondents (n = 83, 47.7%) mentioned details on the duration of care ranging from a few days until more than ten years. The majority of children (n = 52, 62.7%) were cared for up to one year.

### Disposition to commit oneself to palliative medicine

The majority of the paediatricians in this study (75.1%) would be disposed to engage in the palliative care for a child or adolescent (Table 4). Among those, 40.8% said „rather yes", and 34.3% „yes, definitely". Only a very small minority (n = 3; 1.0%) chose the option „no, definitely not". Likewise, a large proportion of the paediatricians would accept different kinds of additional effort for the palliative care. Nearly all of the paediatricians would make home visits (97.3%), prescribe medications under the German narcotic act (95.1%), and participate in further education/training (93.8%). All respondents could imagine asking colleagues for professional advice (100%). Home visits were regarded as reasonable up to a distance of 10 km (range 1 - 80 km).

**Table 4:** Disposition to engage in palliative care for children and adolescents

"Could you generally imagine engaging in the palliative care for a child or adolescent?" (n = 289)			
**No, definitely not** **Rather not**	1.0% 23.9%	→**(rather) not disposed**	24.9%
**Rather yes** **Yes, definitely**	40.8% 34.3%	→**(rather) disposed**	75.1%

**"Which kind of additional effort would you accept for this commitment?" (n = 225)**			

**Home visits at the child's residence**	No Yes	2.7% 97.3%	Range: 1 - 80 km M: 10.4 km (SD 7.4)
**Prescribe medication under the German narcotics act**	No Yes	4.9% 95.1%	
**Ask colleagues for professional advice**	No Yes	0.0% 100.0%	
**Further education/training**	No Yes	6.2% 93.8%	Range: 2 - 140 hours M: 13.9 hrs. (SD 15.5)

**Preferred type**	**1. Self-study**/Internet/Textbooks/Publications	16.0%	
	**2. Discussion group **supervision/case conference	8.6%	
	**3. Internship**/practical instruction/bedside teaching	9.8%	
	**4. Seminar**	30.7%	
	**5. Lecture**	15.3%	
	**7. Quality circle**	7.4%	

The desired number of education hours/year was reported between 10 and 20 hours with a mean of 13.9 hours. Nearly half of the respondents (n = 142, 48.8%) reported on the preferred type of education. Seminars were most frequently mentioned (30.7%), followed by self-study and lectures (15 - 16%).

### Perceived barriers

Generally none of the potential barriers in the questionnaire regarding the implementation of paediatric palliative care stood out in the paediatricians' ratings. Most frequently the time demand was mentioned as a relevant barrier (40.7%), followed by the lack of adequate reimbursement (31.6%). Approximately one third of the respondents evaluated the sole responsibility without staff support (31.1%) as a relevant barrier, as well as the organisational effort related to forms, applications and prescriptions (26.6%). It merits special attention that inhibition in confrontation with death and dying, uncertainty in the contact with patient and family, as well as insecurity regarding the transition from curative to palliative care were only rated by a small proportion of the paediatricians as severe barriers, whereas these factors were evaluated explicitly as *no *barrier by a strikingly high percentage of the respondents (around 70% respectively). For example, inhibition in confrontation with death and dying was rated as a severe barrier only by 10.7%, whereas 26.5% do not perceive this as barrier at all and further 50.9% as an unimportant or small barrier. Likewise, only 19.4% of the respondents rated the emotional burden as a relevant barrier, whereas 2/3 of the paediatricians (66.0%) explicitly evaluated this as no or only a small barrier. However, for those paediatricians who did rate these emotional burdens as major barriers, this seemed to be related to a significant lower disposition to engage in palliative care.

**Figure 2:**
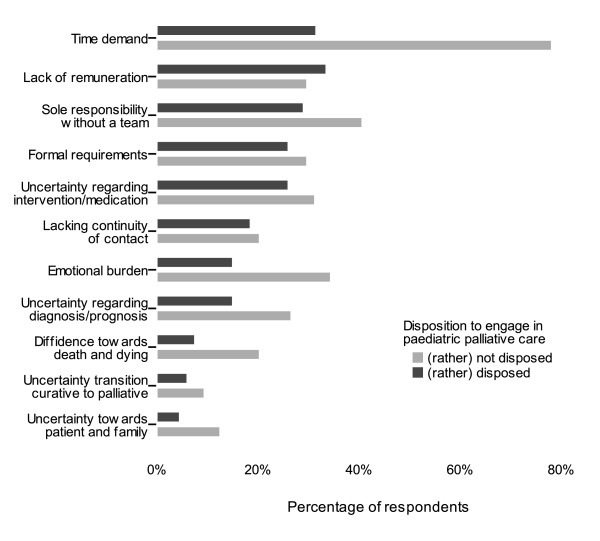
**Barriers in the implementation of paediatric palliative home care**. The barriers (item no. 6 - 16 in the questionnaire) are shown for physicians who (according to their response to question no. 4) were rather disposed to engage in paediatric palliative care (n = 217) compared to those who answered that question in the negative (n = 72).

Overall, meaningful differences were found between those paediatricians who would (rather) commit themselves to palliative care and those who would (rather) not do so (Figure 2). Paediatricians who were principally (rather) not disposed to palliative care rated most barriers clearly more frequently as a relevant obstacle. This discrepancy was particularly evident for the appraisal of the time demand, uncertainties in the handling (contact with patient and family, diagnosis/prognosis, intervention), as well as for the emotional burden and inhibition in confrontation with death and dying.

The physicians' age had no influence on the evaluation of the indicated barriers. Women rated some barriers more frequently as severe compared to their male colleagues. This was most marked for "sole responsibility without a team" (42.9% women vs. 22.8% men), the emotional burden (27.7% women vs. 14.2% men), but also professional uncertainty regarding diagnosis/prognosis (22.4% vs. 14.9%) and inhibition towards death and dying (16.0% vs. 7.5%). In contrast, men evaluated the lack of financial reimbursement more frequently as a relevant barrier (35.4% vs. 25.9%). Paediatricians without prior experience in palliative care rated some barriers more frequently as relevant compared to their colleagues with previous experience: financial burden (38% vs. 25%), emotional burden (24% vs. 13%) and diffidence towards death and dying (15% vs. 5%).

The option of supplemental free-text comments to the pre-indicated barriers was hardly used by the respondents. As an additional barrier the specificity of reimbursement contracts according to the respective health insurance company was deplored.

### Perceived facilitations

According to the paediatricians in this study, the most important facilitations for the implementation of paediatric palliative home care were support by local specialist services (83.0%), availability of a specialist paediatric palliative care consultant or consultation team (82.4%), education in palliative basis competence (60.5%), as well as the option of exchange (for example in a case conference (60.1%)). Education in communication skills was evaluated as relevant facilitation by half of the respondents (48.1%). A 24/7 on-call service for paediatric palliative care was rated as an important incentive by 71.0% of the paediatricians. In additional free-text comments support with a spiritual carer as well as legal safeguarding were mentioned.

**Figure 3:**
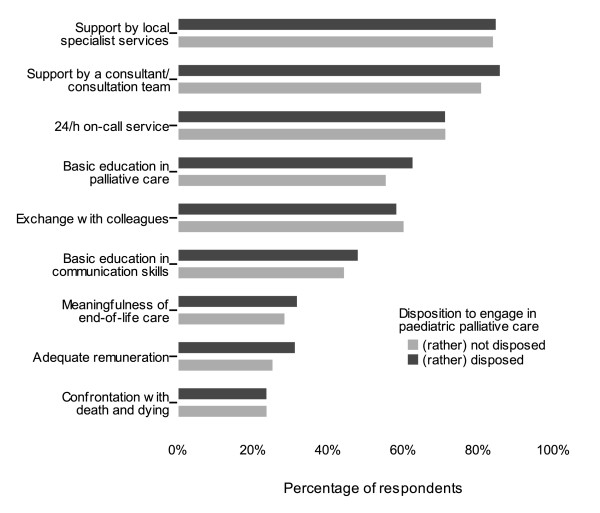
**Facilitations for the implementation of paediatric palliative home care**. The facilitations (item no. 18 - 26 in the questionnaire) are shown for physicians who (according to their response to question no. 4) were rather disposed to engage in paediatric palliative care (n = 217) compared to those who answered that question in the negative (n = 72).

In contrast to the barriers there was no difference in the perception of potential facilitations between paediatricians who were principally disposed to paediatric palliative care and those who were rather not (Figure 3). The respondents' age did not influence the answers, but in analogy to the barriers a slight difference between men and women was found. Men rated the option of an adequate reimbursement more frequently as an incentive (35.5% vs. 27.7%); whereas women more frequently evaluated education in communication skills (54.5% vs. 44.5%) as relevant facilitation, as well as the option of professional exchange (66.4% vs. 56.4%). However, X^2^-tests showed that none of the differences was statistically significant.

### Professional role of the paediatrician with respect to palliative care

The majority of the respondents in this study were of the opinion that paediatricians should have basic knowledge in palliative care (81.8%) and reach a certain degree of confidence and security in this field (80.3%). However, the reimbursement modalities would have to be revised considerably if palliative care delivered by paediatricians in their own practice should be enhanced (74.9%). Likewise the majority (81.1%) agreed with the statement that palliative care should be involved early in the disease trajectory. Half of the respondents (44.9%) believed that the paediatrician should be the central coordinator in the care of children and adolescents with a life-limiting disease. In contrast, one fifth thought that the paediatrician in his own practice only plays a minor role in the care. Two third of the respondents (67.9%) conceded that they were not sufficiently informed about the available specialist paediatric palliative care services in the local proximity. This is especially remarkable since specialist services were regarded as most important facilitation for the implementation of paediatric palliative care.

## Discussion

### Methodological considerations

The paediatricians in this study have been approached via the regional quality circles which have been selected in a systematic way to ensure an equilibrated participation from rural and urban areas, as well as from the two regions North Rhine and Westphalia-Lippe. The demographic and clinical characteristics of the sample confirm that the respondents of this study are a representative selection of paediatricians working in their own practice in North Rhine-Westphalia. Therefore, it may be concluded that the results can be generalised to the total population of paediatricians working in general practice. Social desirability might have played a role since during the survey in the quality circles the investigators (SJ and AV) were present. Regardless of this a considerable proportion of the respondents reported that they were (rather) not disposed to engage in palliative care. This indicates that the questionnaires were not completed in a socially desirable way and that a sufficient reliability of the responses can be assumed. The heterogeneity of answers suggests that the questions were suitable to reveal different views, which endorses the validity of the questionnaire. The option to complete the predefined barriers and facilitations by free-text comments was rarely used, which might be an indication for a good coverage of the relevant aspects by the questions. However, it must be critically acknowledged that the questionnaires were completed during an evening session after a long working day as one topic of a comprehensive agenda, which might have reduced the motivation for further reflection.

### Discussion of the results

The high commitment that was experienced by the researchers during the survey, for example when attending the quality circles, indicates that despite the low case numbers the issue is of high significance for paediatricians in general practice. A remarkable number of paediatricians even answered the questionnaire on their own initiative independent of the social obligation that can be assumed within a quality circle. Also, some respondents wished to complete or illustrate the answers in the questionnaire by anecdotic narrations of their practical experience.

### Experiences with palliative care

The palliative experiences during their professional history reported by the paediatricians emphasise how seldom the care for a child with a life-limiting disease occurs in the general practice - especially when taking into consideration that the respondents in this study were on average 50 years old and had been working in their own practice for a mean duration of 13 years. The heterogeneity of diagnoses underlines the exceptional status of palliative care by a general paediatrician. It seems evident that the required professional competence and the clinical expertise in dealing with these different diseases cannot be sustained within a general paediatric practice.

In this context it must be considered that the frequency of palliative care experiences in this survey presumably was underestimated. Some palliative care experiences might not have been recalled or classified as such by the paediatricians when completing the questionnaire. Physicians may not have been able to recall cases spontaneously. The completion of the questionnaire only lasted shortly and there was no possibility for further reflection or amendments, which suggests that the number of patients has been underestimated. Another reason might be that the paediatrician in charge does not classify the care as "palliative"; either because he is not aware of the disease groups as classified by the ACT or as life expectancy in certain diseases is continuously growing due to innovative therapies. This is underlined by the finding that the most frequently mentioned diagnoses were malignant diseases (group 1 according to the ACT classification), whereas diagnosis of group 2 (e.g. cystic fibrosis, muscular dystrophy) were scarcest.

### Barriers

Overall, it is striking that barriers and obstacles in this study were rated rather reservedly. In the light of the existing deficits in paediatric palliative home care this raises several questions, for example with respect to pain management. Recent studies among general paediatricians (attending community physicians/physicians at a primary medical centre) reported that a large proportion of the respondents felt inexperienced in managing dying patients' symptoms and were not confident in their ability to use narcotics to manage chronic pain [1,2]. However, only one third of the paediatricians in this study report lack of security regarding medical intervention and more than 90% would not hesitate to prescribe narcotics. This does not fit with the observation that there is still a huge reluctance among physicians to prescribe opioids for the treatment of pain. Especially in children and adolescents, the anamnesis, documentation and therapy of pain is evaluated as insufficient [20]. McGrath and Finley in a prior review describe factors for not providing adequate pain medication for children and adolescents [21]. Denial of pain in the child or a downplaying of the harmful effects of pain was reported as major reasons for undertreatment. Moreover, especially in infants the reduced opportunities for verbal communication about symptoms such as pain complicate the assessment. This often leads to an underestimation of the symptom burden in small children [22]. In addition, underestimation may be due to the fact that the quality of care on part of the family is rather attributed to the personal relationship to the attending physician than to objective medical outcomes [23]. Often, neither the physician himself, nor the patient and the family, are well informed about available options to relieve pain and therefore are not aware that the child's pain could be treated more effectively [21]. In consequence, it must be critically questioned whether the responses in this study rather point to a lack of appreciation of the problem. Another reason for the discrepancy between the self rating of the respondents in this survey and the results of former studies might be the wording of the questionnaire. The questions might have been too unspecific to detect true uncertainties (*"uncertainty with regard to intervention and medication" *is less concrete than *"how likely do you rate your ability to use narcotics for the management of chronic pain?"*).

It also merits critical reflection that the majority of paediatricians in this study did not perceive a lack of security on diagnosis and prognosis, as well as the transition from curative to palliative care, as a relevant barrier. Several studies have found that these are the major challenges for health care professionals in paediatric palliative care [7-9].

Likewise, the finding that emotional demands are explicitly reported as being *no burden *or only a *minor burden *must be regarded critically given the evident human challenges inherent to palliative care for children and adolescents. This may be due to the socialisation of (general) paediatricians because the professional self image of physicians even today does not comprise openness towards fears. Schiffman et al. have evaluated the introduction of a paediatric palliative care curriculum for paediatric residents [24]. The curriculum had significant positive outcomes for the residents' self rating of feeling prepared for different skills in the care for a dying child. However, the course had no effects on the belief that "expression of grief is unprofessional" and "residency stress prohibits the processing of and coping with grief". Liben in his review article comes to a similar conclusion [10]: *"grieving of health care professionals remains hidden and disenfranchised, however, because society expects them to remain strong and stoic in the face of death, while institutional regulations strongly discourage the expression of grief"*. This assumption is supported by the finding in our study that women - compared to their male colleagues - more frequently report these issues as relevant barriers, which may be related to higher social acceptance for women to admit emotional fears and burdens.

Several studies in this context raise the concern that emotional barriers towards palliative care, as well as uncertainty regarding the integration of curative and palliative care, can have severe consequences for the referral practice [8,9]. Children with life-limiting diseases suffer from a range of conditions like congenital disorders, neurodegenerative diseases and malignancies where accurate predictions regarding the diagnosis itself and the course of the disease are particularly difficult. Decision making and orientation towards primary treatment goals can be complicated, and bears the risk of a dichotomous thinking in terms of "curative treatment" versus "palliative care" instead of an integrative approach. Despite the current standards and recommendations physicians still lack knowledge or feel reservations to involve specialist palliative care services. Misconceptions or erroneous beliefs can lead to reluctance to refer to palliative care services.

The results of this study raise the question how to bridge the gap between the existing deficits in the provision of paediatric palliative home care and the awareness of the professional challenges by general paediatricians. In this context, the perception of the implementation of paediatric palliative home care from those paediatricians who report that they are *not *disposed to engage in the palliative care of a child or adolescent deserves particular attention. In this group the perception of barriers is quite extensive and includes barriers on different levels (emotional, professional, time demand), implying that a complex approach is required to meet these diverse obstacles. Individual experiences may illustrate the concerns of general paediatricians. For example, one paediatrician described a negative experience in a rural area that would impede him from a future commitment to palliative care. Crucial problems in his view were the organisational effort (long travel distances, adverse weather conditions with ice and snow), as well as the high responsibility that he was not able to share in this rural situation. The paediatrician mentioned that the parents increasingly demanded that he would visit their home. At the same time he experienced a lack of confidence towards his interventions, accompanied by the threat that he would "have the child on his conscience" and could even end up with a law suit if the child's condition would deteriorate, not to mention if the child died. This example underlines the need of a support network for general paediatricians providing palliative home care. This support becomes especially important since the care for children and adolescents with life-limiting diseases can last several years - a much longer duration compared to the care for adults (on average 28 days for patients who are receiving palliative care by general paediatricians in the home care setting) [25].

### Facilitations

In contrast to the barriers the potential facilitations were evaluated more clearly. A high percentage of the respondents consistently rated the predefined aspects as facilitations for the implementation of paediatric palliative home care. The results reflect two major topics: firstly, the importance of support from colleagues, specialists and/or specialist teams in the local proximity. Palliative home care of children and adolescents seems to become more feasible if one does not have to cope with it on his own. Professional and/or practical support by colleagues, local specialist services, availability of a specialist consultant or consultation team, as well as 24/7 on-call service, preferably by a local (children's) inpatient service were wanted. Secondly, education (in basic palliative care competence and in communication skills) was evaluated as helpful. This fitted in with the professional self-image of the paediatricians, as the respondents confirmed that paediatricians in their own practice should attain a certain degree of security and self-confidence in the palliative care of children and adolescents. Similarly, the early involvement of palliative care was clearly supported.

As a rule, the "generalists" (e.g. general practitioners, paediatricians, adult nursing services) will have to take on a major part of the care if coverage of large areas is aimed for. The empowerment of primary carers by consultation and education by paediatric palliative care specialists is crucial in order to establish local networks, especially when taking into account the heterogeneous range of diseases requiring paediatric palliative care. For example, Kelley et al. have developed a customised education programme for the expansion of palliative care delivery in rural areas in Ontario (Canada) with the aim of empowering those carers who do not have specialist palliative care training [26]. Education in the field of paediatric palliative care deserves careful consideration regarding the appropriate form, extent, and specificity of the programme. The study of Schiffman et al. shows that a 6-session paediatric palliative care curriculum for paediatric residents had significant effects on the residents' confidence regarding relevant skills [24]. At the same time, Sheetz & Bowman raised the concern that in the area of paediatric palliative care, advanced training may not be effective nor efficient [2]. Given the low frequency of paediatric deaths in an average general paediatrician's professional life, it will not be possible to sustain skills and knowledge obtained in the training. Furthermore, a new case might confront the paediatrician with completely different palliative care demands than the former patient. The challenge will be to find an education model that conveys basic knowledge, skills, attitudes and a feeling of manageability. In practice, this should ideally be combined with specialist consultation that would provide concrete care instructions for every specific case.

An important work-related coping strategy is seen in the development of professional relationships that promote debriefing and enhance mutual support [10]. Support by specialised local services can share the burden of responsibility. Support by a colleague or a consultation team can raise confidence in acute situations or in medical decision making. The above reported example, as well as free-text answers that were given in addition to the predefined response options in the questionnaire imply that the fear of legal consequences plays an important role. Options for ethical-legal consultation by a specialist centre or team could be included in the counselling services.

In this survey the respondents' rating of the predefined facilitations was not influenced by demographic variables or the general disposition to engage in paediatric palliative care. Facilitations and incentives identified in the survey should apply for the majority of paediatricians and can contribute to a sustainable basis of local palliative care networks. In a further step it might be useful to investigate more systematically which aspects are perceived as barriers and facilitations with regard to specific diagnoses.

## Conclusions

Professional exchange, as well as professional and practical support by colleagues or a consultation team, specialist services in local proximity, as well as a 24/7 on-call service are evaluated as central facilitations for the implementation of paediatric palliative home care by paediatricians in general practice. The need for an adequate financial reimbursement of the additional effort related to palliative home care is emphasised. There is an expressed desire for education in basic competencies in palliative care as well as in communication skills.

The important task will be to address these challenges adequately within the local network structure of services in a certain region.

This means that an effective and efficient combination of basic education, case conferences, and consultation by specialist paediatric palliative care consultants has to be established. Encouraging approaches have been initiated by the specialist centres in North Rhine-Westphalia by offering a multimodal variety of education opportunities comprising a multiprofessional course, case conferences, lecture series, and thematic workshops, as well as specialist consultation and co-treatment.

An important prerequisite for an improved care provision will be the acknowledgement of the professional and emotional demands of this emerging discipline among general paediatricians. It can be assumed that the survey itself has raised a high level of awareness for the subject, and that important information, for example on the existence of the pilot project and the specialist centres for paediatric palliative care, has been conveyed. The underlying research highlights the challenges of the involvement of general paediatricians in the provision of an extensive service delivery in palliative home care for children and adolescents in Germany. Barriers and facilitations are focused from the paediatricians' perspective and are discussed critically in the light of findings described in recent publications originating in other countries, especially the UK and the USA. Therefore, this paper not only reflects issues specific to the German situation, but also contributes to a wider scope of innovative models of paediatric palliative care delivery and to the consolidation of empirically based knowledge in this quite young health care discipline.

## Competing interests

The authors declare that they have no competing interests.

## Authors' contributions

SJ elaborated the methodology of the study including the questionnaire development, managed the data assessment and carried out the expert interviews. She did the qualitative content analysis of the interviews, as well as the descriptive analyses of the quantitative questionnaire results and wrote the article. AV was responsible for the data entry and did the categorisation of free text answers; she critically reviewed the whole research procedure. Both SJ and AV attended the quality circles and handled the distribution of the questionnaires. SM and TF helped to identify issues of relevance for general paediatricians with respect to the provision of palliative care and gave critical feedback to the questionnaire. SM supported the questionnaire development by offering the opportunity of undertaking a pilot test within his regional quality circle. Both SM and TF supported the whole study with their endorsement of the research, as well as the arrangement of contact with regional key persons. BZ contributed to the contacting of appropriate experts for the data assessment and supported the interpretation of data with his specialist expertise in the field of paediatric palliative care. LR supervised the entire study process and gave important advice at all steps of the research project. All authors read and approved the final manuscript.

## Pre-publication history

The pre-publication history for this paper can be accessed here:

http://www.biomedcentral.com/1472-684X/9/11/prepub

## Supplementary Material

Additional file 1 **Survey questionnaire, translated version**. The file contains a translated version of the original German questionnaire used in this survey. Click here for file
