# Supplementary material for: Paediatric palliative home care by general paediatricians: a multimethod study on perceived barriers and incentives
Source: BMC Palliat Care. 2010 Jun 4;9:11. doi: 10.1186/1472-684X-9-11 (PMC2902453; doi:10.1186/1472-684X-9-11)
Supplement: Additional file 1 — Survey questionnaire, translated version. The file contains a translated version of the original German questionnaire used in this survey. [file 1472-684X-9-11-S1.PDF]

Klinik für Palliativmedizin
UNIVERSITÄTSKLINIKUM AACHEN

PAEDIATRIC PALLIATIVE HOME CARE BY GENERAL PAEDIATRICIANS IN THEIR OWN PRACTICE

QUESTIONNAIRE

on behalf of the Ministry of Work, Health and Social Affairs of North Rhine-Westphalia

Ministerium für Arbeit,
Gesundheit und Soziales
des Landes Nordrhein-Westfalen

QUESTIONNAIRE ON PAEDIATRIC PALLIATIVE HOME CARE BY GENERAL PAEDIATRICIANS IN THEIR OWN PRACTICE

The federal state of North Rhine-Westphalia pursues the objective to advance home care for children and adolescents with a severe life-limiting disease. In order to achieve a targeted and sensible improvement of care provision, information has to be gathered about the present care situation. *Your* experiences and *your* point of view are the prerequisite for a further development of care delivery. We ensure that the questionnaires will be dealt with confidently; data processing and analysis will occur anonymously.

In the following questions, *paediatric palliative care* will be used in accordance with the WHO definition and refers to the care for children and adolescents suffering from a life-limiting disease with the aim of improving their quality of life. It begins when illness is diagnosed, and continues regardless of whether or not a child receives treatment directed at the disease. Palliative care can mean the accompaniment of a patient until the end of his life but is not restricted to this phase.

Date |__|__|__|__|__|__|__| **ID-Code |__|__|__|__|__|__|__|**

Prior experience with paediatric palliative home care

1. Do you have experience with palliative care of a child or adolescent in your professional practice?
   ☐ No → proceed to question 4.
   ☐ Yes → proceed to question 2.

2. How many times has this occurred?
   ☐ once
   ☐ < 5 times
   ☐ > 5 times

3. Please describe in short the child's age, diagnosis/es and the duration of care

Disposition to provide paediatric palliative home care

4. Would you generally be disposed to engage (further on) in this field of care, i.e. palliative home care for children and adolescents?
   ☐ Yes, definitely
   ☐ Rather yes
   ☐ Rather not → proceed to question 6.
   ☐ No, definitely not → proceed to question 6.

5. Which kind of additional effort would you accept?

   House calls to the child's home
   ☐ No
   ☐ Yes, up to _______ km

   Prescriptions under the German narcotic act
   ☐ No
   ☐ Yes

   Seek professional advice / consult colleagues
   ☐ No
   ☐ Yes

   Education / training
   ☐ No
   ☐ Yes

   If yes, which type of education? ____

   How many hours per year? ___________

   Other (please specify): ____

Barriers to the implementation

The following items refer to potential barriers that you perceive or have already experienced yourself with respect to the implementation of paediatric palliative home care. Please evaluate the extent to which each of these barriers - according to you - impedes the implementation of care delivery.

0 = does not present a barrier to me
1 = minor barrier to the implementation of care
5 = serious barrier to the implementation of care

		0	1	2	3	4	5
6.	Formalities (forms, applications, prescriptions).	☐	☐	☐	☐	☐	☐
7.	Financial burden (lack of remuneration, settlement systems).	☐	☐	☐	☐	☐	☐
8.	Time demand (travel, working hours, organisation of medical office).	☐	☐	☐	☐	☐	☐
9.	Emotional burden.	☐	☐	☐	☐	☐	☐
10.	Professional uncertainty with respect to diagnosis / prognosis.	☐	☐	☐	☐	☐	☐
11.	Professional uncertainty with respect to appropriate medical care (interventions, medication, etc.).	☐	☐	☐	☐	☐	☐
12.	Diffidence towards death and dying.	☐	☐	☐	☐	☐	☐
13.	Uncertainty towards patient and family.	☐	☐	☐	☐	☐	☐
14.	Uncertainty regarding the transition from *curative* to *palliative*.	☐	☐	☐	☐	☐	☐
15.	Lacking continuity of contact due to temporary treatment of the child / adolescent in specialist outpatient clinics / inpatient units.	☐	☐	☐	☐	☐	☐
16.	Sole responsibility for want of exchange with a team.	☐	☐	☐	☐	☐	☐
17.	Other (please specify): ____	☐	☐	☐	☐	☐	☐

Facilitations to the implementation

The following items refer to types of support that you appreciate as helpful with respect to the palliative home care for children and adolescents. Please evaluate the extent to which each of these options - according to you - facilitates the implementation of care delivery.

0 = does not present a facilitation to me
1 = minor facilitation to the implementation of care
5 = enormous facilitation to the implementation of care

		0	1	2	3	4	5
18.	Availability of a consultant or consultation team to seek advice in case of complicated problems.	☐	☐	☐	☐	☐	☐
19.	Education / training in communication skills.	☐	☐	☐	☐	☐	☐
20.	Meaningfulness of end of life care.	☐	☐	☐	☐	☐	☐
21.	Active reflection on dealing with death and dying.	☐	☐	☐	☐	☐	☐
22.	Adequate remuneration.	☐	☐	☐	☐	☐	☐
23.	Education / training in basic palliative care competence.	☐	☐	☐	☐	☐	☐
24.	Availability of specialist supportive services in local proximity (e.g. paed. nursing home care service, paed. volunteer hospice service).	☐	☐	☐	☐	☐	☐
25.	Opportunity of professional exchange (e.g. in a case conference).	☐	☐	☐	☐	☐	☐
26.	24-h on-call service, preferably provided by (please specify): ____	☐	☐	☐	☐	☐	☐
27.	Other (please specify): ____	☐	☐	☐	☐	☐	☐

The general paediatrician's role in the provision of paediatric palliative home care

The following items refer to the role and the tasks of the general paediatrician in his own practice within paediatric palliative home care. Please evaluate your level of agreement with the subsequent statements.

1 = strongly disagree
5 = strongly agree

	Statement	1	2	3	4	5
28.	General paediatricians should have basic knowledge in palliative care.	☐	☐	☐	☐	☐
29.	Palliative care should be involved at an early stage in the care of children and adolescents with a life-limiting disease.	☐	☐	☐	☐	☐
30.	The general paediatrician merely plays a minor role in the care for children and adolescents with an incurable disease.	☐	☐	☐	☐	☐
31.	General paediatricians should attain a certain degree of certainty and self-confidence in palliative care.	☐	☐	☐	☐	☐
32.	Frequently, general paediatricians are not sufficiently aware of specialist paediatric hospice and palliative care services.	☐	☐	☐	☐	☐
33.	The general paediatrician should be the key contact person for the coordination of palliative care for children and adolescents with a life-limiting disease.	☐	☐	☐	☐	☐
34.	Modalities of remuneration have to be significantly changed in order to promote general paediatricians' engagement in palliative home care for children and adolescents.	☐	☐	☐	☐	☐

Comments and additions

____

____

Demographical data

Personal details: ☐ Man ☐ Woman Age (years) __________

Work details:

Settled in own practice since (year) |__|__|__|__| ☐ own practice ☐ joint practice

Number of patients per quarter ____________ ☐ (rather) rural ☐ (rather) urban

Does your practice have a professional focus? ☐ No ☐ Yes (please specify): ____________________________

Are you engaged in professional policy work? ☐ No ☐ Yes (please specify): ____________________________

WE ARE MUCH OBLIGED TO YOU FOR YOUR SUPPORT!
